# A zinc metabolism-related gene signature for predicting prognosis and characteristics of breast cancer

**DOI:** 10.3389/fimmu.2023.1276280

**Published:** 2024-01-08

**Authors:** Jinghui Hong, Mengxin Li, Yichang Chen, Ye Du, Dong Song

**Affiliations:** Department of Breast Surgery, General Surgery Center, The First Hospital of Jilin University, Changchun, Jilin, China

**Keywords:** zinc metabolism, breast cancer, prognosis, tumour microenvironment, signature

## Abstract

**Background:**

Breast cancer is one of the most serious and prevalent malignancies. Zinc is commonly known to play a crucial role in the development and progression of breast cancer; however, the detailed mechanisms underlying this role are not well understood. This study aimed to develop a zinc metabolism-related gene (ZMRG) signature based on a multi-database study to predict patient prognosis and investigate the relationship between drug therapy response and immune enrichment.

**Methods:**

Data for breast cancer samples from The Cancer Genome Atlas and Gene Expression Omnibus databases were screened for zinc metabolism-related genes using the Molecular Signature Database. Cox and Least Absolute Shrinkage and Selection Operator regressions were performed to construct a ZMRG signature. To assess the predictive performance of the gene signature, Kaplan–Meier analysis and receiver operating characteristic curves were used. Additionally, we utilised single-sample gene set enrichment analysis, the Tumour Immune Estimation Resource, the Genomics of Drug Sensitivity in Cancer database, and the Cancer Therapeutics Response Portal to investigate the association between the tumour microenvironment and drug sensitivity. Quantitative PCR was used to assess the expression of each gene in the signature in breast cancer cell lines and patient samples.

**Results:**

Five ZMRGs were identified (ATP7B, BGLAP, P2RX4, SLC39A11, and TH) and a risk profile was constructed for each. Two risk groups, high- and low-risk, were identified in this way, and the high-risk score subgroups were found to have worse prognosis. This risk profile was validated using the GSE42568 dataset. Tumour microenvironment and drug sensitivity analyses showed that the expression of these five ZMRGs was significantly associated with immune response. The high-risk group showed substantial immune cell infiltration and enrichment of immune pathways, and patients were more sensitive to drugs commonly used in breast cancer.

**Conclusion:**

The ZMRG signature represents a new prognostic predictor for patients with breast cancer, and may also provide new insights into individualised treatment of breast cancer.

## 1 Introduction

Breast cancer (BC), which has been increasing in incidence over the past few decades, is the most prevalent cancer globally and continues to be responsible for a significant number of cancer-related deaths (1). BC can be classified into four molecular subtypes based on estrogen or progesterone receptor expression and Her-2 gene amplification. The different subtypes have different risk profiles and optimal treatment strategies (2). Although the early diagnosis of BC improves survival, a proportion of BC patients still develop metastases after treatment or are diagnosed after metastases have occurred (3). Drug resistance is the central challenge in BC therapy, and it has now been demonstrated that the development of drug resistance is closely linked to the tumour immune microenvironment (TIME) (4).

Zinc (Zn) is an essential trace mineral in the human body and plays a vital role in various biological processes. Zn metabolism relies on two proteins, ZIP and ZNT, for intracellular and extracellular transport of Zn ions (5, 6). Zn acts as a cofactor for more than 300 enzymes and is involved in cell signalling, proliferation, immune function, oxidative stress, and apoptosis (7). Studies have shown that disruption of Zn metabolism contributes to the development of various cancers, including BC, prostate cancer, pancreatic cancer, and hepatocellular carcinoma (8). Zn has also been shown to be involved in the metastasis of BC (9). Patients with BC often exhibit elevated tumour and reduced serum Zn levels (10). Abnormalities in Zn metabolism may directly contribute to BC development and progression. However, the detailed molecular mechanisms underlying the role of Zn in the promotion of tumourigenesis and metastasis requires further exploration. Understanding these mechanisms may provide new insights for breast cancer treatment.

In this study, we aimed to investigate the relationship between Zn and the prognosis and characteristics of BC. We screened RNA expression data from normal and BC tissue samples for differentially expressed Zn metabolism-related genes (ZMRGs) and constructed a prognostic signature based on these ZMRGs. We further analysed the relationship between ZMRGs and the TIME and performed relevant drug sensitivity analysis.

## 2 Materials and methods

### 2.1 Data acquisition and collection

RNA sequencing data and clinical information for BC and normal cases (111 normal and 1033 BC cases) were acquired from The Cancer Genome Atlas (TCGA) data portal (https://portal.gdc.cancer.gov/). The detailed clinical data collected included age, survival time, status, and staging. GSE42568 was used as a validation set and was acquired from the Gene Expression Omnibus (GEO) database (https://www.ncbi.nlm.nih.gov/geo/). ZMRGs were extracted from the Molecular Signatures Database (MSigDB) (https://www.gsea-msigdb.org/gsea/msigdb/).

### 2.2 ZMRG prognostic signature construction and validation

Differentially expressed genes (DEGs) between BC and normal tissues were identified using the “limma” R package. DEGs were identified under the criteria |log2-fold change (FC)| >0.5 and *P* < 0.05 after adjusting for false discovery rate (FDR). Protein–protein interaction (PPI) information for the 76 ZMRGs was obtained from the STRING database (http://www.string-db.org/, version 11.5). We identified the ZMRGs that were significantly associated with prognosis using univariate analysis. Next, we selected stable prognostic genes (R package “glmnet”) using Least Absolute Shrinkage and Selection Operator (LASSO) regression. We constructed a prognostic model from the prognostic ZMRGs using multivariate Cox regression analysis and calculated Zn metabolism-related risk scores (ZMRS) based on the corresponding coefficients. The ZMRG formula was calculated as “Riskscore = gen1 * coef1 + gen2 * coef2 + gen3 * coef3… + genN * coefN” as a specific expression. Samples were divided into high- and low-risk groups based on the median ZMRS score. Time-dependent curve analysis was generated using the “timeROC” R package to assess the predictive accuracy of the prognostic ZMRGs.

### 2.3 Construction and evaluation of the nomogram

Univariate and multivariate Cox regression analyses were conducted to identify the independent prognostic values of the risk scores and of several clinicopathological features (including age, staging, and survival time and status). A prognostic nomogram combining the risk score and the clinical characteristics was constructed using the R package “rms”.

### 2.4 Analysis of correlation with immune status

The enrichment scores of 13 immune-related pathways and 16 immune cell subpopulations were compared between the low- and high-risk groups by single-sample gene set enrichment analysis (ssGSEA) using the R package “gsva”. The Tumour Immune Estimation Resource (TIMER2.0, https://cistrome.shinyapps.io/timer/) was used to obtain information on the expression of ZMRGs and immune cell infiltration. We analysed the relationship between ZMRG expression and the abundance of six types of immune cells: B cells, CD4^+^ T cells, CD8^+^ T cells, neutrophils, macrophages, and dendritic cells (DCs).

### 2.5 Analysis of chemotherapeutic sensitivity and potential drugs in BC

To explore whether the risk prognostic signature is associated with chemotherapy resistance in BC, we used the R package “pRRophetic” to predict the half-maximal inhibitory concentration (IC_50_) of chemotherapeutic agents. Two databases were consulted for potential therapeutic drug screening: the Genomics of Drug Sensitivity in Cancer (GDSC, https://www.cancerrxgene.org/) and the Cancer Therapeutics Response Portal (CTRP, http://portals.broadinstitute.org/ctrp/). From the GDSC database, the IC_50_ and corresponding mRNA gene expression for 265 small molecules from 860 cell lines was collected. This same information was collected for 481 small molecules from 1001 cell lines from the CTRP database. Both datasets were analysed using Pearson correlation analysis to identify the correlation between target gene mRNA expression and drug IC50.

### 2.6 Cell culture

Human breast epithelial (MCF-10A) and BC cell lines (MCF-7, MDA-MB-231, MDA-MB-468, HCC38, SKBR3, BT549, and AU565) were purchased from the American Type Culture Collection (Manassas, VA, USA). The MCF-7, MDA-MB-231, MDA-MB-468, HCC38, and SKBR3 cell lines were cultured in high-glucose DMEM (Hyclone, Logan, UT, USA), whereas BT549 and AU565 cells were cultured in 1640 medium (Hyclone), both containing 10% foetal bovine serum (Cellmax, Lanzhou, China) and 1% penicillin/streptomycin at 37°C, 95% humidity, and 5% CO_2_ in a cell culture incubator.

### 2.7 Quantitative RT-PCR in cell lines and breast tissues

BC and paracancerous tissues from 14 patients with BC were sourced from the First Hospital of Jilin University. Tissue collection was approved by the hospital’s ethics committee. RNA was extracted from the cell lines and breast tissue and was reverse-transcribed to cDNA. Real-time PCR was then performed using the 2 × RealStar Fast SYBR qPCR Mix (GeneStar, Beijing, China) to quantify the transcripts of the five ZMRGs identified as prognostic signatures. Primers were purchased from Jilin Comate Bioscience Co., Ltd. (Jilin, China). The ATP7B, BGLAP, P2RX4, SLC39A11, TH, and GAPDH oligonucleotide primer sequences were: ATP7B (F:GGCCGTCATCACTTATCAGCC; R: GGGAGCCACTTTGCTCTTGA), BGLAP (F:CACTCCTCGCCCTATTGGC; R:CCCTCCTGCTTGGACACAAAG), P2RX4 (F:CTACCAGGAAACTGACTCCGT; R:GGTATCACATAATCCGCCACAT), SLC39A11 (F:CAGCTCTCGTGTTCGTATTCTC; R:TCAGCCAAGTAGACAAAAGCC), TH (F:GCTGGACAAGTGTCATCACCTG; R:CCTGTACTGGAAGGCGATCTCA), GAPDH (F:GGAGCGAGATCCCTCCAAAAT; R: GGCTGTTGTCATACTTCTCATGG). Target gene expression was quantified relative to the internal control, GAPDH.

### 2.8 Statistical analysis

All statistical analyses were performed using R software (version 4.3.0). The Log-Rank test was used to detect survival rates in each group, the Wilcoxon test was used to compare the differences between the two data groups, and the Kaplan–Meier method was used to plot the survival curves of patients in both groups. Univariate Cox regression and multivariate Cox analysis were used to construct the prognostic signatures and assess their value. *P <* 0.05 was considered statistically significant.

## 3 Results

### 3.1 Identification of ZMRGs and DEGs in BC

**Figure 1:**
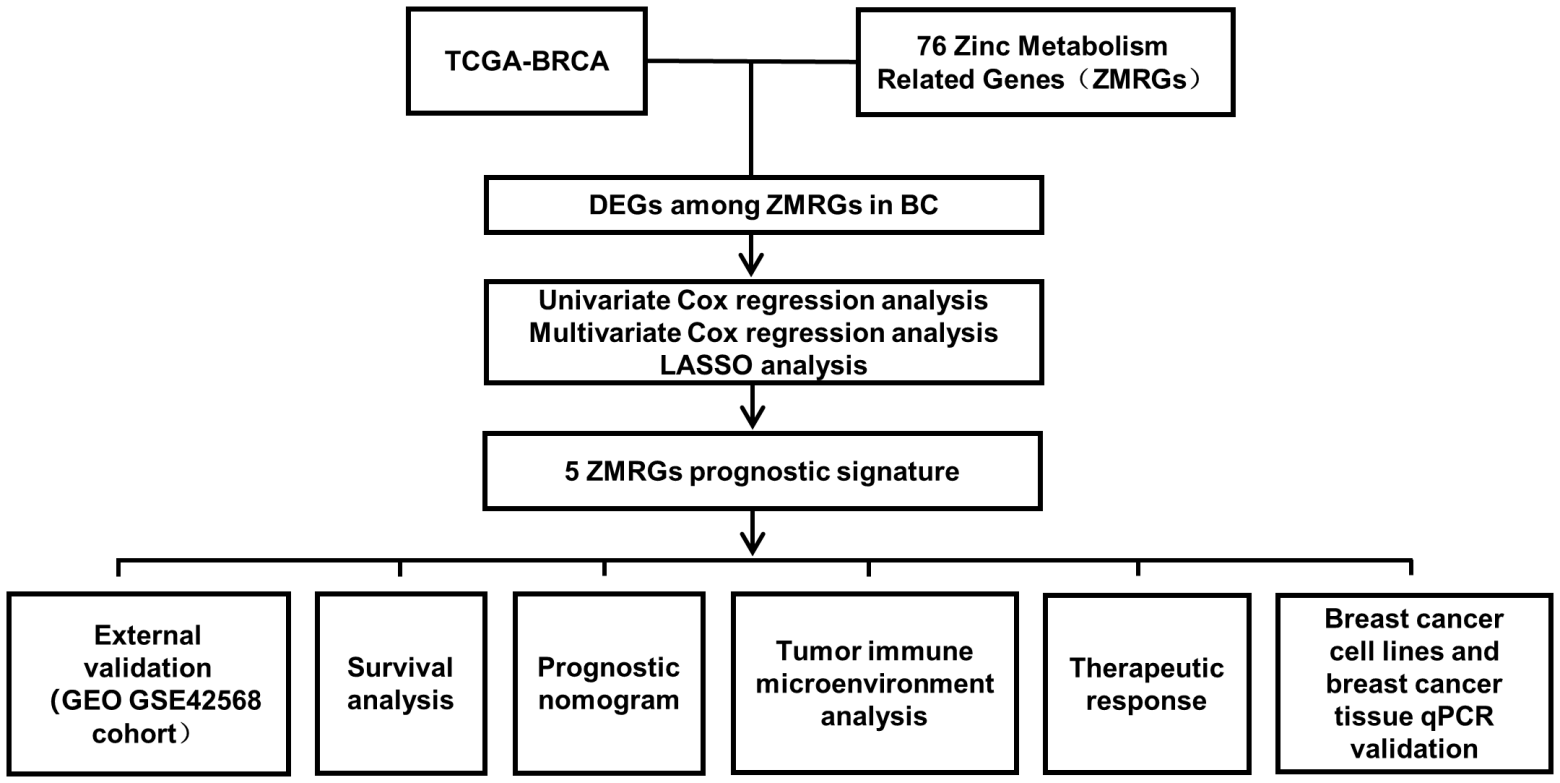
Workflow diagram of this study.

**Figure 2:**
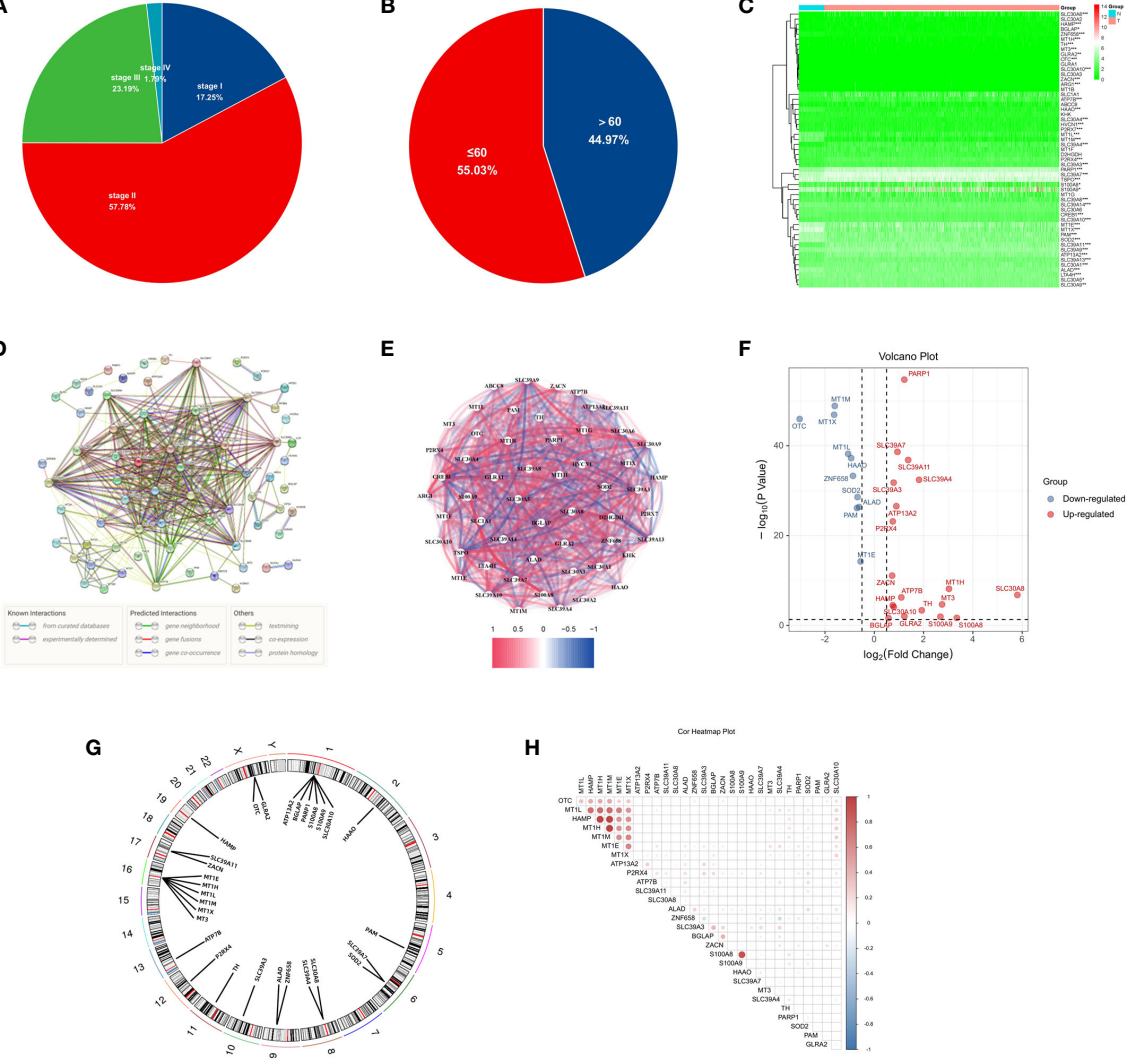
Expression and Genetic characteristics of ZMRGs in TCGA-BRCA cohort. **(A)** Age distribution in the TCGA-BRCA cohort. **(B)** Tumour stage distribution in the TCGA-BRCA cohort. **(C)** Heatmap of 76 differentially expressed ZMRGs. **(D)** PPI analysis among the 76 ZMRGs. **(E)** The correlation network of the ZMRGs. **(F)** Volcano plot illustration of differentially expressed ZMRGs in the TCGA-BRCA cohort. **(G)** The location of the ZMRGs on chromosomes. **(H)** Correlation matrix among the ZMRGs.

The workflow diagram of this study is shown in **Figure 1**. The age and tumour stage differences across the cases analysed in this study are shown in **Figures 2A**, **B**. To pinpoint genes related to Zn metabolism, we chose multiple pathways using the MSigDB database and identified 76 related genes (**Figure 2C**). To further explore the interactions of the selected ZMRGs, we performed a PPI analysis (**Figure 2D**). We set the minimum interaction score required for the PPI analysis to 0.9 (highest confidence level). The correlation network containing all the ZMRGs is shown in **Figure 2E**. In addition, we found 29 DEGs by comparing gene expression patterns in 1033 BC samples with those in 111 normal breast samples using thresholds |log2FC| > 0.5 and FDR < 0.05. Among these DEGs, 19 were upregulated and 10 were downregulated in BC (**Figure 2F**). The location on the chromosome and the expression levels of the 29 DEGs are illustrated in a circular plot (**Figure 2G**). The features of association between these genes were revealed using correlation matrix plots (**Figure 2H**).

### 3.2 Development of a ZMRG prognostic signature

**Figure 3:**
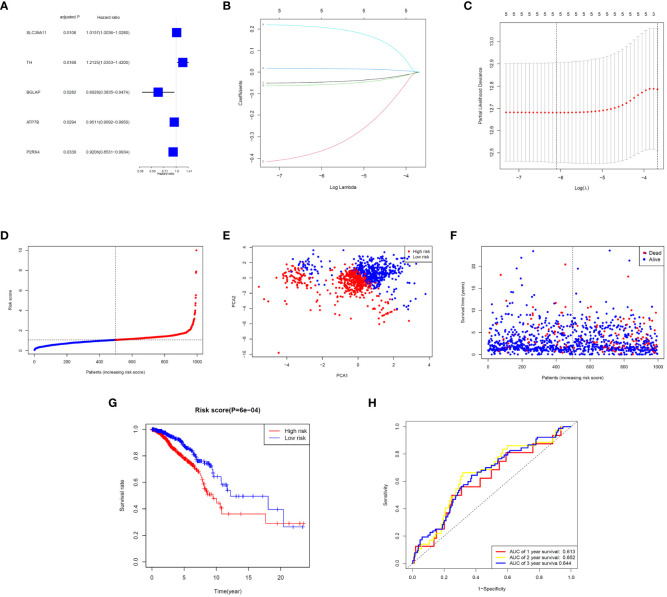
Correlation analysis between prognosis and characteristics of BC patients from the TCGA-BRCA database. **(A)** Univariate Cox regression of the five ZMRGs. **(B)** LASSO regression of the five ZMRGs. **(C)** Cross-validation in the LASSO regression. **(D)**. The distribution of risk scores of the five ZMRGs. **(E)** The PCA plots based on risk scores. **(F)** The distribution of OS status of the five ZMRGs. **(G)** Kaplan–Meier survival curves for OS in the two risk groups. **(H)** AUC values of ROC curves for risk scores.

Five significant prognostic ZMRGs were identified using univariate Cox regression in the TCGA-BC cohort (**Figure 3A**). All 29 DEGs were screened for prognostic value using LASSO regression (**Figures 3B, C**). Subsequently, the corresponding coefficient values were extracted and individual risk scores were calculated based on the coefficient-weighted expression levels of the selected genes. The risk score was calculated as follows: risk score = (–0.052542 × ATP7B expression) + (–0.436158 × BGLAP expression) + (–0.065372 × P2RX4 expression) + (0.017788 × SLC39A11 expression) + (0.223976 × TH expression). Patients were stratified into high- and low-risk groups according to the median cut-off values (**Figure 3D**). Principal component analysis (PCA) demonstrated that samples were clearly divided into two groups according to their risk scores (**Figure 3E**). The distributions of the risk scores and survival metrics are shown in **Figure 3F**. With the increase in risk score, the risk of death increased, while the time to overall survival (OS) decreased. Kaplan–Meier survival curve analysis revealed that patients in the high-risk group had significantly worse OS than those in the low-risk group (*P* < 0.001; **Figure 3G**). Time-dependent ROC curve analysis was used to estimate the predictive power of the prognostic ZMRGs (**Figure 3H**). The AUCs were 0.613 and 0.644 for the ZMRGs at 1 and 3 years of follow-up, respectively, indicating relatively good predictive accuracy.

**Figure 4:**
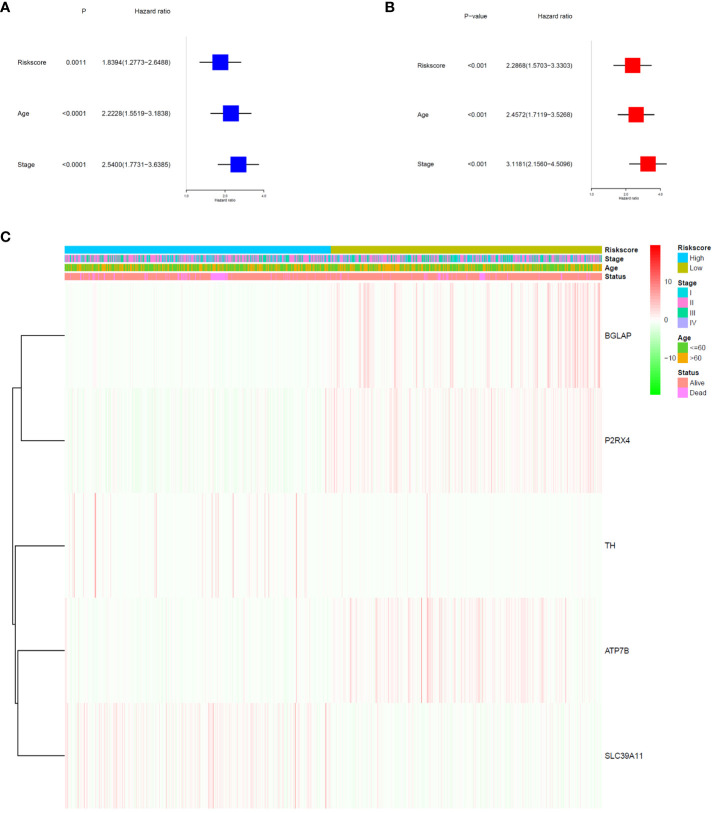
Prognostic value of the risk signature. **(A)** Univariate Cox analysis on OS for the risk score in TCGA-BRCA cohort. **(B)** Multivariate Cox regression on OS for the risk score in TCGA-BRCA cohort. **(C)** Heatmap of the association between clinical and pathological features in the two risk groups.

The signature significantly predicted survival in univariate and multivariate Cox regression analyses. Univariate Cox regression analysis indicated that the risk score was an independent predictor of poor survival in patients with BC (HR = 1.8394, 95% CI: 1.2773–2.6488; **Figure 4A**). Multifactorial analysis yielded the same result, with the risk score being identified as an independent prognostic factor (HR = 2.2868, 95% CI: 1.5703–3.3303; **Figure 4B**) after adjusting for other clinical characteristics, including age and tumour stage. In addition, we generated a heatmap of the clinical characteristics of TCGA cohort (**Figure 4C**).

### 3.3 Validation of the ZMRGs signature in GEO dataset

**Figure 5:**
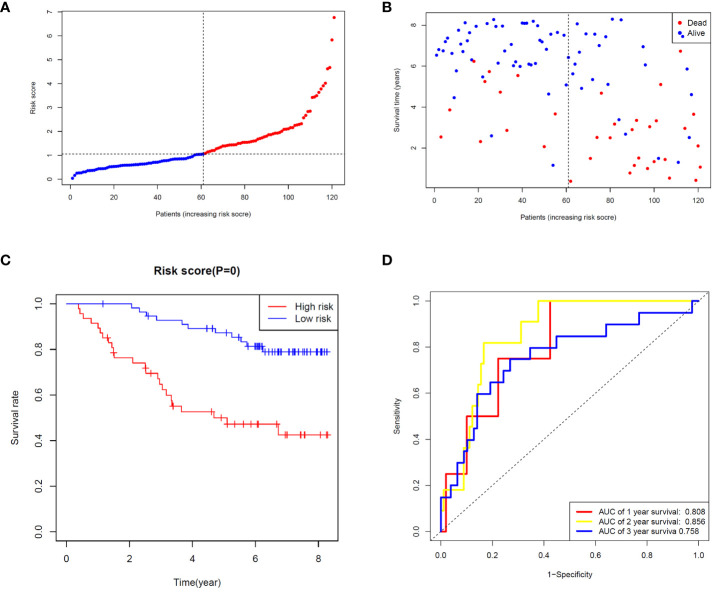
Validation of risk characteristics in the GEO cohort using ZMRG risk scores. **(A)** The distribution and median risk score in the GEO cohort. **(B)** PCA distribution. **(C)** Kaplan–Meier survival curves for OS in the two risk groups. **(D)** AUC values of ROC curves for risk scores in the GEO cohort.

To validate the ability of the selected ZMRG signature to predict BC prognosis, we selected the external cohort GSE42568 (104 BC samples) from the GEO database. The risk model estimated risk scores for selected patients were calculated using the same formula and the same cut-off values were used to classify them as high- or low-risk patients (**Figure 5A**). Similar to the results obtained from the original TCGA cohort, patients in the high-risk group were more likely to die (**Figure 5B**). Survival analysis revealed significantly different survival rates between the two risk groups (**Figure 5C**). The AUCs were 0.808 and 0.758 for the ZMRGs at 1 and 3 years of follow-up, respectively, meaning we also achieved good predictive accuracy in the GEO cohort (**Figure 5D**).

### 3.4 Evaluation of a clinicopathologic nomogram

**Figure 6:**
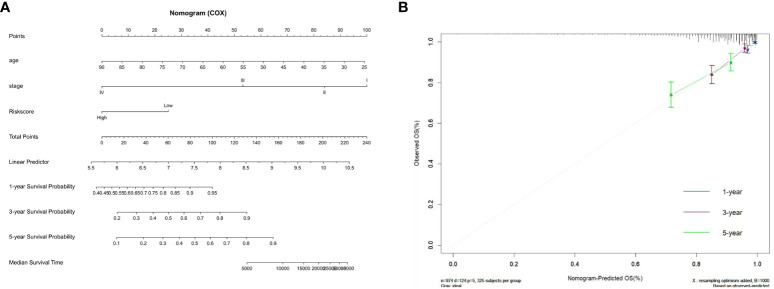
Development of the prognostic nomogram. **(A)** Nomogram based on the five ZMRGs comprising the signature and the clinical information. **(B)** The calibration plots for predicting 1-, 3-, and 5-year survival probabilities.

We constructed a nomogram by combining the ZMRG signature with two clinical characteristics (age and AJCC stage) to better predict 1-, 3-, and 5-year OS in patients with BC (**Figure 6A**). The plotted calibration curves showed a good agreement between the predicted and actual survival curves, confirming that the column line graphs had satisfactory predictive discrimination (**Figure 6B**).

### 3.5 Association between immune status/tumour immune infiltration and risk score of ZMRGs

**Figure 7:**
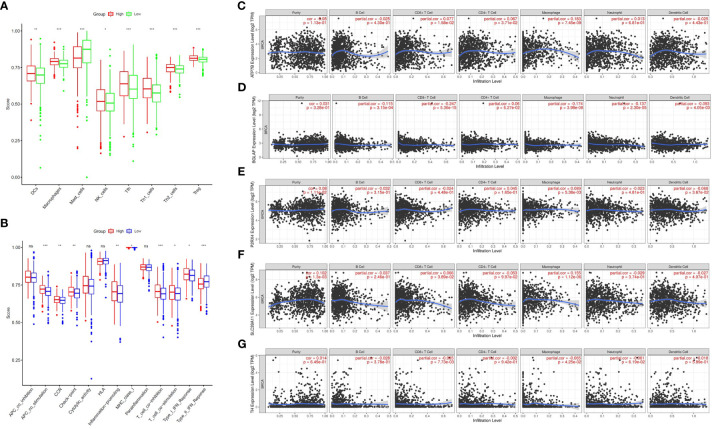
Comparison of ssGSEA scores between the two risk groups and correlation between the expression of the five ZMRGs and immune infiltration in BC. **(A)** Enrichment scores for eight immune cell types in the two risk groups. **(B)** Enrichment scores for 13 immune-related pathways in the two risk groups. ns, not significant; *P < 0.05; **P < 0.01; ***P < 0.001. **(C–G)** Correlation between ATP7B, BGLAP, P2RX4, SLC39A11, TH expression and immune infiltration in BC based on the TIMER database.

Zn metabolism plays a significant role in breast tumour immunity. To further investigate the association between the ZMRG risk scores and tumour immunity, the enrichment scores of 16 immune cell subpopulations and 13 immune-related pathways were compared between the low- and high-risk groups by single-sample gene set enrichment analysis (ssGSEA). The results showed that eight immune cell types (DCs, macrophages, mast cells, NK cells, Tfhs, Th1 cells, Th2 cells, and Tregs) were significantly more enriched (*P* < 0.05) in the high-risk group than the low-risk group, meaning higher levels of immune cell infiltration were present in the high-risk group (**Figure 7A**). In terms of immune-related pathways, nine pathways showed significantly higher (*P* < 0.05) immune activity in the high-risk group than in the low-risk group (**Figure 7B**). Next, the correlation between the expression of the five prognostic ZMRGs (ATP7B, BGLAP, P2RX4, SLC39A11, and TH) and the level of BC immune infiltration was explored. The results suggested that the expression of these ZMRGs was associated with infiltration patterns of many immune cells. The expression level of ATP7B was positively correlated with the level of immune infiltration of CD8^+^ T cells (*P* = 1.58 × 10^–2^), CD4^+^ T cells (*P* = 3.71 × 10^-2^), and macrophages (*P* = 7.45 × 10^–9^) (**Figure 7C**). The expression level of BGLAP was positively correlated with the level of immune infiltration of B cells (*P* = 3.15 × 10^–4^), CD8^+^ T cells (*P* = 5.36 × 10^–15^), macrophages (*P* = 3.98 × 10^–8^), neutrophils (*P* = 2.30 × 10^–5^), and dendritic cells (*P* = 4.05 × 10^–3^) were negatively correlated with the level of immune infiltration (**Figure 7D**). The level of P2RX4 expression positively correlated with the level of immune infiltration of macrophages (*P* = 5.38 × 10^–3^) and negatively correlated with the level of immune infiltration of dendritic cells (*P* = 3.67 × 10^–2^) (**Figure 7E**). The expression level of SLC39A11 was positively correlated with the level of immune infiltration of CD8+ T cells (*P* = 3.89 × 10^–2^) and macrophages (*P* = 1.12 × 10^–6^) (**Figure 7F**). The expression level of TH was positively correlated with the level of immune infiltration of CD8^+^ T cells (*P* = 7.73 × 10^–3^) and was negatively correlated with the level of infiltration of macrophages (*P* = 4.25 × 10^–2^) (**Figure 7G**). To further validate the role of ZMRGs in the biological process of immunity, we performed a GO enrichment analysis of DEGs between the high-risk and low-risk groups. The results showed that ZMRGs were significantly enriched in the biological processes of immunization, consistent with the results of this part of the study (**Supplementary Figure 1**).

### 3.6 Exploration of chemotherapeutic response and potential drugs for BC based on ZMRGs

**Figure 8:**
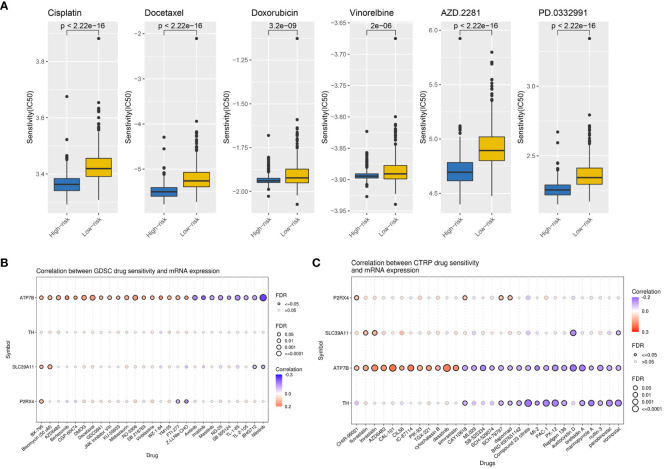
Potential therapeutic agents for BC based on ZMRG risk scores. **(A)** Comparison of IC_50_ values for commonly used drugs recommended in BC treatment guidelines in two risk groups. **(B)** Drug sensitivity analysis based on the GDSC database, drugs such as BX-795 and bleomycin may be therapeutically effective. **(C)** Drug sensitivity analysis based on the CTRP database, drugs such as CHIR-99021, fluvastatin, and lovastatin may be therapeutically effective. Positive correlations are represented by red bubbles and negative correlations by blue bubbles. The darker the colour of the bubbles the higher the correlation. The size of the bubbles is positively correlated with the significance of FDR. A black border on the bubbles indicates FDR ≤ 0.05.

To explore the value of the ZMRG-related risk scores for predicting common chemotherapeutic agents for BC, we used the “pRRophetic” database to predict the chemotherapeutic responses in BC patients. We calculated the IC_50_ values of commonly used drugs in the high- and low-risk groups. The IC_50_ values of drugs recommended in the current major BC treatment guidelines include cisplatin, docetaxel, doxorubicin, vinorelbine, palbociclib (PD.0332991), and olaparib (AZD2281), among others, were lower in the high-risk group than in the low-risk group, meaning there was greater sensitivity to these drugs in the high-risk group (**Figure 8A**). We also identified several potential BC therapeutics using the GDSC and CTRP databases. According to the GDSC database analysis, BX-795, bleomycin, and others may be effective drugs (**Figure 8B**). Based on analysis of the CTRP database, CHIR-99021, fluvastatin, lovastatin, and others may be effective (**Figure 8C**).

### 3.7 Validation of the mRNA expression level of ZMRGs

**Figure 9:**
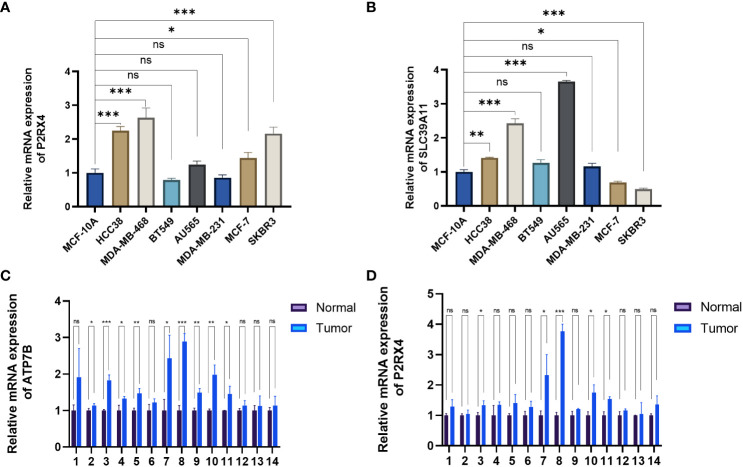
Validation of mRNA expression levels of the five ZMRGs. **(A, B)** The mRNA expression level of P2RX4 and SLC39A11in breast cancer cell line, as measured by qRT-PCR. **(C, D)** The mRNA expressions of ATP7B and P2RX4 in breast tissue as measured by qRT-PCR. *P < 0.05; **P < 0.01; ***P < 0.001; ns, no significance.

The expression of the ZMRGs which form the signature was validated using qRT-PCR. We detected gene expression in several common breast cancer cell lines and a normal breast epithelial cell line, as well as comparing expression between breast cancer and paracancerous tissues from 14 patients. The results showed that P2RX4 and SLC39A11 were highly expressed in most breast cancer cell lines (**Figures 9A, B**), and ATP7B and P2RX4 were significantly up-regulated in BC tissues (*P* < 0.05) (**Figures 9C, D**), which is consistent with the results of previous bioinformatics analyses based on the TCGA database and other results described in the literature.

## 4 Discussion

In recent years, the relationship between BC and Zn has received increasing attention. Zn is an important trace element in the body and is involved in regulating numerous cellular metabolic processes. Abnormal Zn metabolism can lead to the development and progression of BC (11). However, studies on the prognostic value of genes related to Zn metabolism in BC have not yet been reported. Prognostic signatures are essential for precision medicine and can help guide clinical decisions. Therefore, we explored the ability of ZMRGs to predict prognosis and established a prognostic signature to provide more accurate therapeutic decisions and diagnostic approaches for BC.

Our prognostic signature included five ZMRGs (ATP7B, BGLAP, P2RX4, SLC39A11, and TH). Previous studies have explored the relationship between ZMRGs and tumours, with all ZMGRGs studied being found to play a role in tumour development and progression. ATP7B is highly expressed in many tumours including BC, ovarian cancer, oesophageal cancer, gastric cancer, and hepatocellular carcinoma (12, 13). ATP7B is not only involved in tumour progression, but also in the development of platinum drug resistance (14–16). A previous study reported that BGLAP was associated with advanced breast cancer staging (17). P2RX4 belongs to the P2 purinergic receptor family, which is commonly upregulated in a variety of tumours and is associated with poor prognosis (18). Specifically, P2RX4 plays a key role as a regulator in inflammation and immune cell function (19). A previous study showed that P2RX4 was upregulated in BC samples, which is largely similar to our findings. It has also been demonstrated that P2RX4 enhances BC invasiveness, breast tumour growth, and metastasis (20). Given its role in promoting tumour formation, P2RX4 has emerged as a potential therapeutic target (21). SLC39A11 is a solute carrier of membrane transport protein family 39 (SLC39A). SLC39A11 was previously found to be significantly upregulated in BC tissues compared to normal breast tissues (22), which is similar to our findings. In colon cancer, SLC39A11 expression is also upregulated, possibly in response to the increased Zn demand in cancer cells (23). SLC39A11 is also a prognostic indicator and therapeutic target in lung adenocarcinoma (24). TH encodes a protein that is involved in the conversion of tyrosine to dopamine. Higher levels of TH expression are associated with worse outcomes, and positive TH expression is a poor prognostic indicator of metastatic neuroblastoma (25).

It is well known that Zn is important for immune function and that abnormalities in Zn metabolism affect immune cells and lead to alterations in host defences (26). Zn is involved in the regulation of multiple intracellular signalling pathways in innate and adaptive immune cells (27, 28). In innate immunity, Zn deficiency affects the activity of natural killer cells and phagocytosis by macrophages (8, 29). In adaptive immunity, some Zn transporter proteins that are highly expressed in T cells, such as Zip6 and Zip8, are directly involved in T cell activation via Zn endocytosis (30). Additionally, Zn is an essential cofactor that directly affects the biological activity of thymosin, which in turn affects T-cell generation, maturation, differentiation, proliferation, and functional changes (31, 32). Our ssGSEA results confirmed the presence of large immune cell infiltration and enrichment of immune pathways in the high-risk group, suggesting that more immune pathways were activated in the high-risk group than in the low-risk group. These results suggest that the high-risk group is generally immunologically activated, indicating that patients with a high-risk signature are more likely to show a better response to immunotherapy, which may be a focus for future therapeutic development.

Interactions between different components of the tumour microenvironment affect tumour development and metastasis and promote BC chemoresistance (33). Chemoresistance during BC treatment significantly affects prognosis. The mechanism of chemoresistance in tumours is complex and involves interactions between numerous signalling pathways (34). Clarifying the mechanisms underlying drug resistance is challenging. Therefore, it is crucial to accurately differentiate drug-sensitive populations. In our study, drug sensitivity analysis was performed on high- and low-risk groups, defined using our ZMRG signature, drawn from the GDSC and CTRP databases. High-risk patients were found to be more sensitive to drugs commonly used in BC, such as cisplatin, docetaxel, doxorubicin, vinorelbine, palbociclib, and olaparib. Using our ZMRG prediction model to evaluate the sensitivity of BC drug therapy, we hope to provide new therapeutic ideas for BC treatment and more precise and individualised treatments for patients with BC in future clinical practice.

Despite improving confidence in our results through our multifaceted analyses, database validation, and validation of multiple BC cell lines and patient samples, this study has some limitations. First, this was a retrospective study, and data for all patients with BC were collected from public databases. Second, further validation of our findings is needed in future studies because the drug sensitivity prediction conducted here is based only on bioinformatics analyses. In addition, the molecular mechanisms underlying the function of the ZMRGs which contribute to our risk score in BC need to be explored in more depth.

In summary, we constructed a prognostic signature based on ZMRGs to predict the prognosis of BC patients. This model is related to the immune microenvironment and can distinguish the immune status of patients with BC. More importantly, it could be used to provide new treatment options for BC patients in different risk groups.

## Data availability statement

The raw data supporting the conclusions of this article will be made available by the authors, without undue reservation.

## Ethics statement

The studies involving humans were approved by Ethics Committee of the First Hospital of Jilin University. The studies were conducted in accordance with the local legislation and institutional requirements. The participants provided their written informed consent to participate in this study.

## Author contributions

JH: Data curation, Conceptualization, Project administration, Validation, Writing – original draft, Writing – review & editing, Methodology. ML: Validation, Software, Writing – review & editing. YC: Software, Investigation. YD: Methodology, Writing – review & editing. DS: Supervision, Funding acquisition, Methodology, Writing – review & editing.
