# Supplementary figures and images for: A zinc metabolism-related gene signature for predicting prognosis and characteristics of breast cancer
Source: Front Immunol. 2024 Jan 8;14:1276280. doi: 10.3389/fimmu.2023.1276280 (PMC10800782; doi:10.3389/fimmu.2023.1276280)

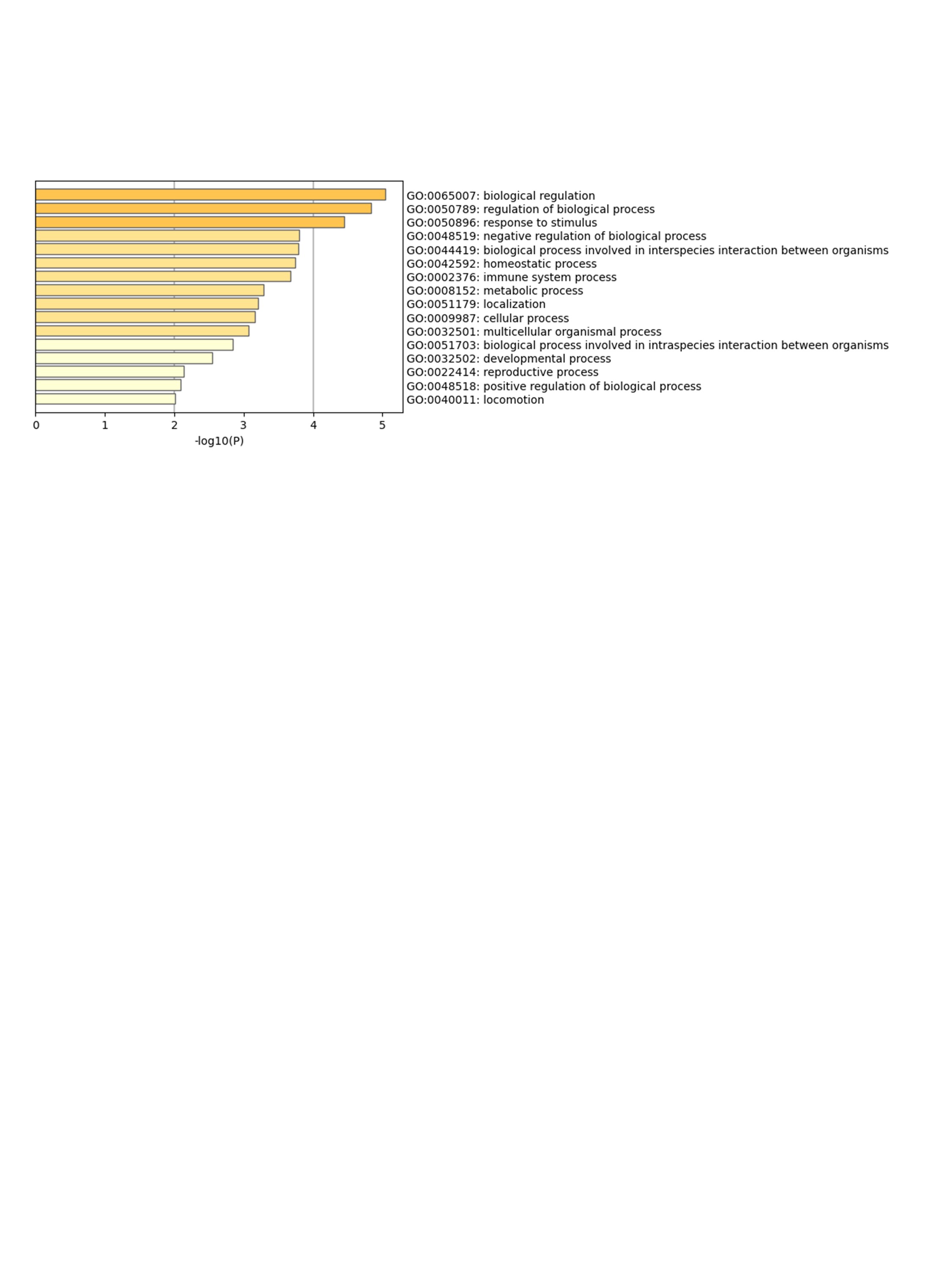

Supplement: Supplementary file 1 [file Image_1.jpg]
